# The Influence of Workplace Envy on Employees’ Knowledge-Hiding Behavior Based on a Comparative Analysis between Generation Cohorts

**DOI:** 10.3390/bs13090716

**Published:** 2023-08-28

**Authors:** Xiaoyan Su, Chufu Chen

**Affiliations:** International Business School, Jinan University, Zhuhai 519070, China; tsuxyan@jnu.edu.cn

**Keywords:** knowledge hiding, benign envy, malicious envy, promotion regulatory focus, prevention regulatory focus, generation cohorts

## Abstract

How to promote the free flow of knowledge among employees is the core factor used to improve the innovation ability and even competitive advantage of an organization. Research on how to reduce knowledge-hiding behavior and promote knowledge sharing among employees becomes the key to enhancing the technological innovation capability of enterprises and effectively responding to the VUCA environment at present. Based on social comparison theory and regulatory focus theory, this study uses 402 enterprise employees as samples to deeply study the influence mechanism of workplace envy on their knowledge-hiding behavior and compare the differences between new-generation employees and non-new-generation employees. The research results show that: (1) employee’s benign envy has a significant negative effect on knowledge-hiding behavior, while malicious envy has a significant positive effect on knowledge-hiding behavior. (2) Promotion regulatory focus plays a partly mediating effect between benign envy and employee knowledge-hiding behavior, while prevention regulatory focus also plays a partly mediating effect between malicious envy and employee knowledge-hiding behavior. (3) Generation not only has a positive moderating effect on the relationship between benign envy and promotion regulatory focus but also has a positive moderating effect on the relationship between malicious envy and prevention regulatory focus. This study further found that the younger the employee generation cohort, the more substantial the effect of benign envy on the promotion regulatory focus, while the older the employee generation cohort, the more substantial the effect of malicious envy on the prevention regulatory focus.

## 1. Introduction

In a knowledge-based economy, knowledge serves as a critical resource for enterprises to secure a sustainable competitive advantage [1]. Establishing an environment conducive to knowledge sharing and fostering knowledge creation by urging employees to disseminate their expertise is vital for effective knowledge management [2]. Given that a substantial portion of an organization’s knowledge resides within individual employees, harnessing this collective expertise and bolstering knowledge exchange among members can significantly augment organizational knowledge stock and innovation capability [3]. In practical enterprise management, while many organizations implement policies to encourage knowledge sharing internally, there is an observable reluctance among employees to share or, worse, deliberately conceal their knowledge. This tendency can evolve into a counterproductive norm, with evidence suggesting that deliberate knowledge hiding is pervasive [4]. For instance, a survey found that 46% of respondents would choose not to share knowledge with colleagues upon request [5]. Another study by AMR indicated that despite substantial investments to foster knowledge sharing, knowledge retention remains prevalent [6]. Such deliberate withholding, termed “knowledge hiding”, may momentarily benefit the individual but, in the long haul, it could undermine the performance at individual, team, and organizational levels. It is also detrimental to cultivating a knowledge-sharing culture and inhibiting innovation. Hence, addressing knowledge hiding to bolster knowledge sharing is crucial for enhancing enterprises’ technological innovation capabilities, especially in the volatile, uncertain, complex, and ambiguous (VUCA) environment [7]. Concurrently, delving into the ramifications of knowledge hiding has enriched associated theoretical discussions, advancing academic insights into knowledge sharing within organizational behavior.

The extant literature on employees’ knowledge-hiding behavior primarily revolves around three central themes: personal factors, organizational and leadership factors, and the characteristics of the knowledge itself. Focusing on personal factors, a body of research has explored the interplay between the Big Five personality traits and knowledge hiding. Studies by Lin and Wang [8], Anand and Jain [9], and Wen et al. [10] found that extraversion, neuroticism, and agreeableness were negatively correlated with knowledge-hiding tendencies. In contrast, conscientiousness and openness were positively associated with such behaviors [11]. Another study illuminated a pronounced positive correlation between employees’ psychological ownership of knowledge and knowledge hiding [12]. Furthermore, workplace rejection was shown to enhance knowledge-hiding tendencies. On the organizational and leadership front, research has underscored the negative influence of a conducive organizational knowledge-sharing environment, trust atmosphere, and equity atmosphere on knowledge-hiding tendencies [13,14]. Leadership styles also play a pivotal role. For instance, shared leadership [15] and non-abusive leadership dynamics [16] act as deterrents to knowledge-hiding behaviors among employees. Turning to the characteristics of knowledge, its complexity, uniqueness, and significance are found to bolster knowledge-hiding tendencies among employees [17]. Collectively, these comprehensive research findings enrich our comprehension of the intricacies within the knowledge-hiding domain.

Within organizational contexts, workplace envy has emerged as a prevalent emotion among employees. This is primarily attributable to finite resources, propelling incessant competition for opportunities such as promotions, pay hikes, and career advancement [18,19]. Rooted in social comparison, workplace envy arises when employees make upward comparisons, leading to painful emotional experiences. Such envy can engender feelings of inferiority, hostility, and resentment, prompting individuals to engage in potentially destructive behaviors in an attempt to bridge perceived disparities [20]. Despite its significance, the literature offers limited insight into the influence of workplace envy on knowledge-hiding behaviors. Initial studies were primarily concentrated on the detrimental aspects, particularly malicious envy. Research has elucidated that malicious envy can instigate workplace ostracism and curtail employees’ proclivity for self-improvement [21]. However, recent scholarly endeavors have shed light on a constructive facet of workplace envy, termed “benign envy” [22,23]. Although both malicious and benign envy stem from social comparisons, they differ inherently, guiding individuals toward markedly distinct behavioral paths. While malicious envy propels individuals toward actions detrimental to organizational well-being, benign envy acts as a catalyst, motivating individuals to augment organizational performance through heightened dedication [24]. Yet, the realm of benign envy remains under-explored. Specifically, there is a dearth of studies that delineate the two dimensions of workplace envy and investigate their distinct impacts on knowledge-hiding behaviors.

Social comparison theory posits that in the absence of objective benchmarks, individuals engage in self-evaluative comparisons with others to bolster their self-esteem and self-worth [25,26]. Festinger [24] asserts that social comparison is an intentional act, with individuals typically selecting peers resembling themselves, such as colleagues or classmates, for comparison. The outcomes of these comparisons significantly sway subsequent attitudes and behaviors. Envy emerges as the disconcerting emotion experienced when individuals discern that others possess what they desire [27]. Recognizing this disparity, individuals might undertake measures to bridge the perceived gap, thereby alleviating their internal dissonance [28,29]. This paper proceeds to delve into regulatory focus theory [30], offering a nuanced examination of how various dimensions of workplace envy influence knowledge-hiding behaviors. Furthermore, this research juxtaposes the effects of workplace envy on knowledge hiding across different generational cohorts. This comparison is pivotal, considering the pronounced divergences in values and behavioral patterns among employees of different age groups, which are shaped by their unique socio-economic experiences [31].

This study delves into the intricate mechanisms underlying the impact of workplace envy on employees’ knowledge-hiding behaviors, drawing from both social comparison theory and regulatory focus theory. We propose a dual-path model that distinguishes the influence of two dimensions of workplace envy: benign envy and malicious envy. This study seeks to elucidate the inner mechanisms of how these dimensions shape knowledge-hiding behaviors and aims to validate the roles of promotion regulatory focus and prevention regulatory focus therein. Furthermore, anchoring our exploration in the generation cohort theory, we investigate intergenerational variations in the effects of workplace envy on knowledge-hiding behavior. This aspect aims to discern the distinct responses of varied generational cohorts in analogous situations. Next, this paper is organized as follows: the second part is the literature review and hypotheses; the third part is the research design, including aspects of sample selection and variable measurement; the fourth part is the analysis of empirical results; and the final part is the research results and discussion.

### 1.1. Literature Review and Hypotheses

#### 1.1.1. Workplace Envy and Knowledge-Hiding Behavior

Research on workplace envy is predominantly anchored in social comparison theory, which posits that individuals contrast their capabilities and attributes with others to derive a distinct self-evaluation [11]. Given the inherent competition for limited resources in the workplace, employees invariably engage in comparisons with peers, especially when vying for such scarce resources. Consequently, workplace envy surfaces as an emotional byproduct of these comparisons within a competitive milieu [32]. Historically, the literature predominantly associated workplace envy with counterproductive employee behaviors, such as hostility and sabotage. However, recent scholarship has acknowledged the duality of envy, bifurcating it into “benign” and “malicious” facets [33,34]. For workplace envy, we divide it by whether it will generate hostility toward the comparison object. Benign envy, devoid of hostility, encompasses emotions of desire without animosity toward the envied. It serves as a catalyst, driving individuals to earnestly pursue their aspirations. Conversely, malicious envy—laden with hostility—harbors resentment toward the envied and impels individuals toward actions aimed at diminishing this discomforting emotion. The divergent motivations emanating from these envy types are palpable: benign envy propels employees to relentlessly hone their skills and chase their ambitions, while malicious envy incites detrimental behaviors intended to undermine the comparison target [35,36,37].

Within organizational contexts, the competitive landscape often seeds envy among employees. This envy, born from upward social comparisons, propels individuals to behaviors that mitigate the discomfort associated with such comparisons and bolster a positive self-view. Given that knowledge stands as a pivotal resource enabling employees to accrue organizational power and status, many opt to expand their knowledge base as a tactic to reinforce their stature within the organization. When faced with a colleague’s request for knowledge sharing, maliciously envious employees, driven by resentment, may resort to knowledge hiding. Their aim? To curtail the colleague’s comparative advantage and bridge the perceived competence gap [25,38]. Conversely, employees harboring benign envy, while recognizing their positional disparities, align with the spirit of collective advancement. Such employees are predisposed to sharing knowledge, envisaging mutual learning and enhancement as the payoff. Existing literature corroborates these dynamics: malicious envy often fuels exclusionary behaviors aimed at stunting peers’ professional trajectories, whereas benign envy serves as a catalyst, spurring individuals to amplify collaborative endeavors and personal achievements [39,40]. As a result, this paper proposes the following hypotheses.

**Hypothesis** **1a:** *Employee benign envy negatively influences knowledge-hiding behavior.*

**Hypothesis** **1b:** *Employee malicious envy positively influences knowledge-hiding behavior.*

#### 1.1.2. The Mediating Effect of the Regulatory Focus

The regulatory focus theory, introduced by Higgins [41], posits that individuals’ self-regulatory orientations lead them to adopt distinct behaviors and strategies in the pursuit of goals. Broadly, this theory discerns between two primary self-regulatory focuses: promotion and prevention [42]. Those with a promotional regulatory focus on prioritizing personal growth and development, aiming for positive outcomes. They are oriented toward achieving aspirations and the embodiment of their “ideal self”. Conversely, individuals with a prevention regulatory focus emphasize personal safety and security. Their behaviors typically skew toward avoidance, as they are keen to circumvent negative outcomes or potential losses. They prioritize fulfilling responsibilities and becoming their “ought self”, ensuring they do not deviate from expected roles or norms [43,44].

Thus, drawing from the regulatory focus theory, it can be posited that the nature of envy—whether benign or malicious—can activate distinct regulatory focus responses in individuals. Benign envy, being rooted in a positive emotional space, is likely to stimulate the promotion focus in employees. When experiencing benign envy, employees feel inspired by the success or qualities of the comparator and are often motivated to elevate themselves to a similar stature or accomplishment. Such envy serves as a catalyst, driving individuals to aspire, learn, and achieve more, aligning with the characteristics of the promotion focus where individuals seek growth and opportunities. Conversely, malicious envy, stemming from a negative emotional standpoint, can trigger the prevention focus in employees. This form of envy is characterized by resentment toward the comparator and is often paired with a desire to see them lose or face setbacks. Rather than self-improvement, the emotions fuelled by malicious envy incline employees toward defensive actions, safeguarding their current status, or even engaging in behaviors to undercut the comparator. Such responses reflect the essence of the prevention focus, wherein individuals are more cautious, risk-averse, and intent on avoiding adverse outcomes. In light of the above, it becomes evident that the emotional undertone of envy—whether positive (benign) or negative (malicious)—plays a pivotal role in determining the regulatory focus response it elicits in individuals, thereby influencing their consequent behaviors and strategies in the workplace. As a result, the following hypotheses are proposed in this paper.

**Hypothesis** **2a:** *Employees’ benign envy has a positive effect on promotion regulatory focus*.

**Hypothesis** **2b:** *Employees’ malicious envy has a positive effect on prevention regulatory focus*.

Promotion regulatory focus pertains to individuals’ growth and development needs. Consequently, employees with this focus aim to achieve their ideal selves, working to bridge the discrepancy between their current and ideal states. When seeking knowledge, such employees are inclined to enhance their skills and understanding through knowledge sharing and communication and are driven by personal growth motivations [45]. Conversely, prevention regulatory focus emphasizes individuals’ needs for security and stability. Employees under this focus strive to attain their desired selves, seeking to minimize the distance between their current state and this ideal. Within a knowledge-seeking context, those with a prevention regulatory focus are apprehensive about potential losses of power and status and the increasing gap with peers. As a result, they prioritize knowledge protection and security, often displaying knowledge-hiding tendencies and limiting their sharing [46,47]. As a result, this paper proposes the following hypotheses.

**Hypothesis** **3a:** *Promotion regulatory focus reduces employees’ knowledge-hiding behavior.*

**Hypothesis** **3b:** *Prevention regulatory focus increases employees’ knowledge-hiding behavior*.

Integrating Hypothesis 2 and Hypothesis 3 reveals that upward social comparisons among employees, leading to workplace envy, foster two distinct regulatory focus tendencies. These tendencies subsequently influence knowledge-hiding behaviors differently. Specifically, benign envy, characterized as a positive emotion, activates the promotion regulatory focus in employees, reducing knowledge-hiding tendencies. In contrast, malicious envy, rooted in negative emotionality, triggers the prevention regulatory focus, increasing the likelihood of knowledge hiding [11,31]. As a result, the following hypotheses are proposed in this paper.

**Hypothesis** **4a:** *Promotion regulatory focus plays a mediating effect in the relationship between benign envy and knowledge-hiding behavior*.

**Hypothesis** **4b:** *Prevention regulatory focus plays a mediating effect in the relationship between malicious envy and knowledge-hiding behavior*.

#### 1.1.3. The Moderating Effect of Generation

In recent times, the influx of newer generations such as the post-1980s, post-1990s, and post-2000s into the workforce has accentuated inter-generational diversity. Consequently, the concept of generation cohorts has garnered significant attention in human resource management discourse. Mannheim defines a generation cohort as a group born within the same timeframe and shaped by identical socio-economic, political, and cultural influences [48]. These cohorts, experiencing pivotal historical events during formative years, develop shared generational identities and consciousness. This shared experience gives rise to common values, cognitive frameworks, and behavioral tendencies. It is also evident that generational groups, molded by varied socio-historical contexts, possess distinct psychological attributes and preferences [49,50].

Research on generational classification in Europe and the U.S. primarily delineates four groups: the Traditionalists (born pre-World War II), Baby Boomers (1946–1964), Generation X (1965–1980), and Generation Y (post-1980), the latter often termed the “new generation”. Meanwhile, China’s generational divisions are more varied, with frameworks ranging from two to four generational cohorts [51,52,53]. Using strict birth years as classification criteria oversimplifies distinctions. For instance, employees born in December 1979 and January 1980 have experienced virtually the same societal context, with no significant differences. Based on an extensive review of the literature and the significance of China’s 1992 economic landmark, this study categorizes current working generations into two broad cohorts: the new generation (born post-1992) and the non-new generation (pre-1992). This division stems from China’s pivot toward a market-driven economy in 1992 [54]. In October 1992, the pivotal 14th Party Congress asserted China’s commitment to a socialist market economy, marking a departure from planning-driven resource allocation. This transition reinvigorated state-owned enterprises, clarifying property rights and responsibilities, and emphasizing the “separation of government and enterprises” and “scientific management”. Consequently, state-owned enterprises gained momentum, and the private sector became instrumental in employment, taxation, and technological innovation. The new generation, molded in this renewed economic landscape, typically boasts better education, coupled with heightened learning aptitude and innovative spirit [55,56].

While a wealth of research has explored inter-generational differences in areas such as citizenship behavior, innovation behavior, consumer behavior, and employee well-being [53,57], the relationship between workplace envy and knowledge-hiding behavior across different generations remains under-investigated. Much of the prevailing literature leverages Meyer et al.’s [58] work values framework to discern generational distinctions, focusing on three primary areas: competence and growth, status and independence, and comfort and security. These values, respectively, reflect individuals’ emphasis on learning opportunities and factors like income and promotion and the perceived safety of the work environment. Contrastingly, the new generation, which matured amidst China’s post-reform rapid socio-economic transformation, tends to have a pronounced inclination toward competence and growth. They frequently seek growth via continual learning and communication. On the other hand, the non-new generation (e.g., post-1960s and post-1970s cohorts) was raised during the more materially constrained planned economy, manifesting values centered on income, achievement, and authority. Empirical findings corroborate that the new-generation employees lean more toward competence and growth values, whereas the non-new-generation inclines toward status and independence [59].

In organizational contexts, generational distinctions might influence how workplace envy affects knowledge hiding, which stems from the varying values of different generational cohorts. While non-new-generation employees often prioritize preserving their existing achievements and status, their newer counterparts emphasize competence and growth in their work value orientation. Consequently, when experiencing benign envy, new-generation employees are likely to manifest a more pronounced promotion regulatory focus. This disposition decreases knowledge-hiding tendencies, fosters knowledge sharing, and bolsters collective competence and knowledge acquisition alongside peers. Conversely, in situations of malicious envy, non-new-generation employees, relative to their newer peers, display a heightened prevention regulatory focus. Such an inclination amplifies knowledge hiding and curtails knowledge sharing. Individuals are motivated by a desire to safeguard their present income and achievements. As a result, this paper proposes the following hypotheses.

**Hypothesis** **5a:** *“Generation” positively moderated the relationship between benign envy and promotion focus. The younger the employee’s generation, the more pronounced the effect of benign envy on the promotion regulatory focus*.

**Hypothesis** **5b:** *“Generation” positively moderated the relationship between malicious envy and prevention regulatory focus. The older the employee’s generation, the more pronounced the effect of malicious envy on the prevention regulatory focus.*

**Figure 1:**
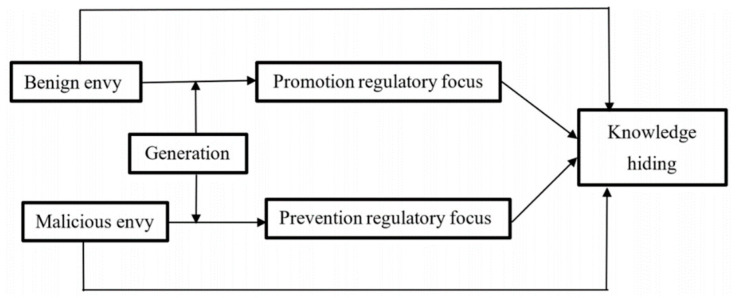
The theoretical model.

In summary, the theoretical model proposed in this study is shown in Figure 1.

## 2. Research Design

### 2.1. Sample and Data Collection

This study used a questionnaire survey to collect data. A non-probabilistic method was used to select a convenience sample. In order to avoid the problem of common method bias, the anonymity of the respondents was protected, and the reverse scoring method was used in this study. Our questionnaire collection was divided into two phases to collect two samples of data, and the interval between the two phases was about half a year. In the first stage, the data samples were mainly distributed and collected on-site for working MBA students at a university in South China. In the second stage, our questionnaire was mainly commissioned to the questionnaire company for questionnaire collection, in which the completed users were also restricted to the South China region. Out of the total 429 questionnaires collected, 402 valid questionnaires were obtained by excluding the questionnaires that were not filled out or filled out indiscriminately, and the effective recovery rate was 93.71%. Table 1 shows the basic statistical information of the surveyed respondents. In the survey sample, 44.03% are male employees and 55.97% are female employees; 53.48% are new-generation employees and 46.52% are non-new-generation employees; 39.30% of the total sample are managers and 60.70% are ordinary employees; 17.91% are employees with 3 years of experience or less, 35.32% are have worked for 4–10 years, and 46.77% have worked for more than 10 years; and 20.65% of the employees have postgraduate education or above, 63.92% have bachelor’s degree, and 15.42% have specialist education.

### 2.2. Measurement

The measurement instruments adopted in this study are all proven scales used in domestic and international studies with good reliability and validity and have also been validated in the Chinese context. Combined with the subjects of this study, the expressions in the relevant scales were appropriately adjusted to make the scale entries more accurately expressed. In this study, the Likert five-point scale was used, with “1” to “5” indicating “Strongly disagree” to “Strongly agree”.

Workplace envy, the envy scale developed by Lange and Crusius [60] (Appendix A, Table A1), was used. It consists of two dimensions, benign envy and malicious envy, with five items each. It includes benign envy items, such as “If I find that my colleagues are better than me, I will try to make myself better”, and malicious envy items, such as “Seeing other colleagues’ outstanding achievements makes me resentful”. Cronbach’s alpha reliability coefficients for the benign envy and malicious envy scales were 0.931 and 0.873, respectively.

Knowledge-hiding behavior, the knowledge-hiding scale developed by Connelly et al. [14] containing 12 questions was selected. For example, “I promised to help but did not actually act on the part for which other members were responsible” and “I informed that my specialty was not in this area”. In this study, Cronbach’s alpha coefficient for the knowledge-hiding behavior scale was 0.961.

Regulatory focus, the work regulatory focus scale developed by Neubert et al. [61], was selected and consists of eighteen items, including two dimensions and promotion regulatory focus and prevention regulatory focus, with nine items each. Promotion regulatory focus is the idea that “At work, I want to take advantage of opportunities to maximize my career advancement goals”, and prevention regulatory focus is the idea that “At work, I focus on completing the duties assigned to me”. Cronbach’s alpha reliability coefficients for the promotion and prevention focus scales were 0.913 and 0.899, respectively.

Generation is represented by a dummy variable, where “1” represents the “new generation” (born in 1992 and later) and “0” represents the “non-new generation” (born before 1992).

Control variables, referring to previous related studies, such as gender, job class, years of work, and education level, can have an effect on the outcome variables. Therefore, the above demographic variables were used as control variables in this study.

### 2.3. Analysis of Study Results

#### 2.3.1. Common Method Biases Test

First, Harman’s one-way test was used in this study to test the common method biases problem of the questionnaire data. The results from the principal component analysis showed that the variance explained by the first principal component variable was 38. 119%, which was lower than 40% and met the basic requirements of the study.

Then, we used the control unmeasured common potential latent method with relatively good testing power. We used AMOS21.0 to construct a two-factor model based on the original trait factor model (a validated factor analysis model consisting of benign envy, malicious envy, promotion regulatory focus, prevention regulatory focus, and knowledge hiding) by adding common method bias and compared the two-factor model with the original trait factor model. The comparison was conducted. The results showed that the fit of the two-factor model was not significantly improved. CFI < 0.001, TLI < 0.001, RMSMA < 0.001, and SRMR < 0.001 met the criteria of CFI and TLI < 0.1; RMSMA and SRMR < 0.05. Thus, the common method biases in this study are not a serious problem.

#### 2.3.2. Discriminant Validity Test

In this study, the discriminant validity of the five latent variables of benign envy, malicious envy, promotion regulatory focus, prevention regulatory focus, and knowledge hiding was tested using AMOS 21.0, and the fit effect was compared with other competing models (four-factor, three-factor, and one-factor models) using the measurement model (five-factor model). As shown in Table 2, the five-factor model fit indices were all within the acceptable range and significantly outperformed the fits of the other competing models (χ2/df = 2.233, RMSMA = 0.055, CFI = 0.926, TLI = 0.921, IFI = 0.926, SRMR = 0.053). This indicates that these five latent variables have good discriminant validity and belong to different constructs.

As shown in Table 3, the CR values of benign envy, malicious envy, promotion regulatory focus, prevention regulatory focus, and knowledge hiding are 0.934, 0.874, 0.915, 0.9, and 0.962, respectively, which meet the criteria of being greater than 0.7. This further indicates that the reliability of the variables is good. The AVE values were 0.741, 0.582, 0.548, 0.502, and 0.677, respectively, which met the criterion of being greater than 0.5, indicating that the construct validity of the variables was good.

#### 2.3.3. Descriptive Statistical Analysis

In this study, descriptive statistics were analyzed for each variable using SPSS 25.0, and the means, standard deviations, and correlation coefficients for each variable are shown in Table 4. In Table 4, it can be seen that benign envy was significantly negatively correlated with knowledge-hiding behavior (r = −0.371, *p* < 0.001), benign envy was significantly positively correlated with promotion regulatory focus (r = −0.587, *p <* 0.001), the promotion regulatory focus was significantly negatively correlated with knowledge-hiding behavior (r = 0.358, *p* < 0.001), malicious envy had a significant positive correlation (r = 0.519, *p* < 0.001) with prevention regulatory focus (r = 0.519, *p* < 0.001), and prevention regulatory focus was significantly positively correlated with knowledge-hiding behavior (r = 0.403, *p* < 0.001). The results of the above correlation analysis provided a good basis for the next test. 

## 3. Hypothesis Testing

### 3.1. Main and Mediating Effects Test

This paper uses multiple regression analysis for hypothesis testing, and the results are shown in Table 5 and Table 6. In model 1, it can be seen that benign envy has a significant negative effect on employees’ knowledge-hiding behavior (β = −0.359, *p* < 0.001), and Hypothesis 1a holds. In model 2, it can be seen that benign envy has a significant positive effect on promotion regulatory focus (β = 0.596, *p* < 0.001), and Hypothesis 2a was confirmed. Model 3 shows that promotion regulatory focus has a significant negative effect on knowledge-hiding behavior (β = −0.358, *p* < 0.001), and Hypothesis 3a was confirmed. Model 4 shows that after adding the mediating variable promotion regulatory focus to model 1, the coefficient of the effect of benign envy on knowledge-hiding behavior is −0.227 (*p* < 0.05) and still significant, indicating that promotion regulatory focus plays a partly mediating effect between benign envy and knowledge-hiding behavior; therefore, Hypothesis 4a was confirmed.

Model 5 shows that malicious envy has a significant positive effect on employee knowledge-hiding behavior (β = 0.337, *p* < 0.001), and Hypothesis 1b was confirmed. Model 6 shows that malicious envy has a significant positive effect on prevention regulatory focus (β = 0.521, *p* < 0.001), and Hypothesis 2b was confirmed. Model 7 shows that prevention regulatory focus has a significant positive effect on knowledge hiding (β = 0.389, *p* < 0.001). Model 8 shows that the positive effect of malicious envy on knowledge-hiding behavior is 0.183 (*p* < 0.01) and is still significant after adding the mediating variable prevention regulatory focus to model 5, indicating that prevention regulatory focus plays a partly mediating effect between malicious envy and knowledge-hiding behavior; therefore, Hypothesis 4b was confirmed.

In this paper, the mediating effect was also further tested using the PROCESS plug-in function of SPSS software based on the Bootstrap method proposed by Chen et al. [62]. The bias-corrected nonparametric percentile method was used, and Bootstrap was set to repeat sampling 5000 times at a confidence level of 95%. In the test results in Table 7, we can learn that the mediating effect value of promotion regulatory focus is −0.132 with a 95% confidence interval [−0.213, −0.063], which does not contain 0, indicating that the mediating effect is significant, and Hypothesis 4a is again verified; that is, benign envy triggers employees’ tendency to promotion regulatory focus, which in turn inhibits the generation of knowledge-hiding behavior and the mediating effect of prevention regulatory focus. The value of 0.154, with a 95% confidence interval [0.095, 0.225], does not contain 0, indicating a significant mediating effect, and Hypothesis 4b is again tested; that is, malicious envy triggers employees’ tendency toward prevention regulatory focus, which in turn promotes the production of knowledge-hiding behavior.

### 3.2. Moderating Effects Test

Next, this paper will test whether the effect of workplace envy on knowledge-hiding behavior differs across generational groups of employees. According to Wen et al. [63], if the independent variable is a continuous variable and the moderating variable is a categorical variable, a group regression should be applied in the test before a comparative analysis of whether there is a significant difference in the moderating effect is performed. In this study, since the independent variables of benign envy and malicious envy are continuous variables and the moderating variables are categorical variables, the study first distinguished between the “new generation” and “non-new generation” groups and processed the data in the following steps In the first step, four control variables including gender, job class, years of working and education level were added to the regression equation. In the second step, the main effects of benign envy and malicious envy on knowledge hiding were added to the regression equation. In the third step, the correlation coefficients of benign envy and promotion regulatory focus and the correlation coefficients of malicious envy and prevention regulatory focus were transformed into Fisher’s z values, and a z-test was conducted to analyze whether there was a significant difference between the relationship between workplace envy and regulatory focus in the two groups and to determine whether there were inter- generational differences in the above moderating effect.

As shown in Table 8, the regression coefficient of benign envy on promotion regulatory focus was 0.483 (*p* < 0.001) when the independent variable of benign envy was added to the “non-New Generation” group. Compared to model 9, ΔR^2^ increased by 21.2% in model 10, while the regression coefficient of benign envy on the promotion regulatory focus was 0.666 (*p* < 0.001) when the independent variable of benign envy was added to the “new generation” group and ΔR^2^ increased by 47.5% in model 11 compared to model 12. The Z-test revealed a significant difference in the explanatory power of benign envy for the promotion regulatory focus between the “new generation” and “non-new generation” groups (Z = 2.75, *p* < 0.01). Since the explanatory power of benign envy in the “new generation” group was much higher than the “non-new generation” group, the correlation coefficient between benign envy and promotion regulatory focus was greater in the new generation group (r = 0.662) than in the non-new generation group (r = 0.477). Therefore, it can be judged that generation has a significant moderating effect on the relationship between benign envy and promotion regulatory focus; therefore, the younger the employee generation, the more significant the effect of benign envy on promotion regulatory focus. Thus, Hypothesis 5a was supported.

Similarly, as shown in Table 9, the regression coefficient of malicious envy on the prevention regulatory focus was 0.661 (*p* < 0.001) when the independent variable malicious envy was added to the “non-new generation” group. Compared to model 13, the ΔR^2^ in model 14 increased by 37.8%, indicating that the explanatory power of the model has improved, while the regression coefficient of malicious envy on the prevention regulatory focus was 0.432 (*p* < 0.001) after adding the independent variable malicious envy in the “new generation” group. Compared to model 15, the ΔR^2^ coefficient in model 16 increased by 19.1%, indicating a slight increase in the explanatory power of the model. The Z-test revealed a significant difference in the explanatory power of malicious envy on the prevention regulatory focus between the “new generation” and “non-new generation” cohorts (Z = 3.57, *p* < 0.001). The explanatory power of malicious envy for prevention regulatory focus was much higher in the “non-new generation” group than the “new generation” group, and the correlation coefficient between malicious envy and prevention regulatory focus was much higher in the “non-new generation” group than in the “new generation” group. The correlation coefficient between malicious envy and prevention regulatory focus (r = 0.675) is significantly higher than the “new generation” group (r = 0.430); therefore, it can be seen that “generation” has a significant moderating effect on the relationship between malicious envy and prevention regulatory focus. The older the employee’s generation, the more significant the effect of malicious envy on the prevention regulatory focus. Hypothesis 5b is supported.

**Figure 2:**
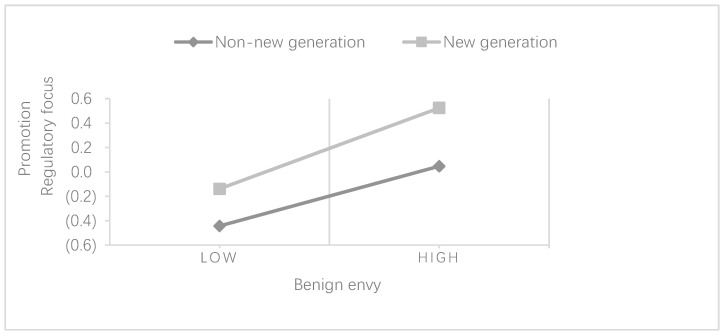
The moderating effect of generation on the relationship between benign envy and promotion regulatory focus.

**Figure 3:**
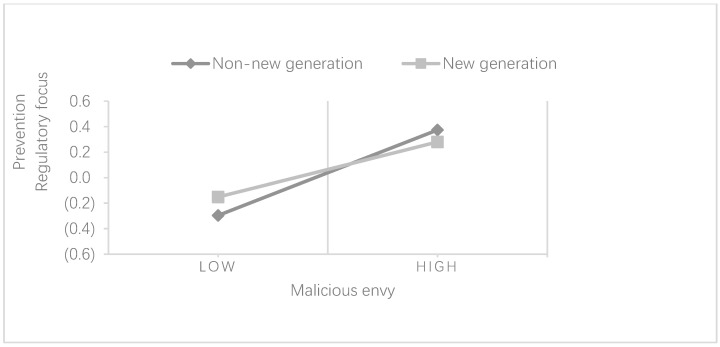
The moderating effect of generation on the relationship between malicious envy and prevention regulatory focus.

In the analysis of Table 8 and Table 9 above, it can be seen that there is a significant generational difference in the effect of workplace envy on the regulatory focus, indicating that “generation” has a significant moderating effect on the relationship between workplace envy and the regulatory focus (Figure 2 and Figure 3).

## 4. Research Results

Drawing upon the foundations of social comparison and regulatory focus theories, this study, encompassing a sample of 402 enterprise employees, devises a model to examine the influence of workplace envy on knowledge-hiding behavior. The research aimed to understand the unique impacts of benign and malicious envy on knowledge hiding and discern inter-generational variations in these effects.

First, employees’ benign envy has a significant negative effect on their knowledge-hiding behavior, while malicious envy has a significant positive effect on their knowledge-hiding behavior. That is to say, all the relevant content of Hypothesis 1 can be demonstrated.

Second, the regulatory focus plays a partly mediating effect between workplace envy and knowledge-hiding behavior. Specifically, employees’ benign envy significantly and positively influenced their promotion regulatory focus, which had a negative effect on knowledge-hiding behavior, and the promotion regulatory focus had a partially mediating role between benign envy and knowledge-hiding behavior. Employees’ malicious envy significantly and positively influenced their prevention regulatory focus and the prevention regulatory focus positively influenced knowledge-hiding behavior, and the prevention regulatory focus partially mediated the relationship between malicious envy and knowledge-hiding behavior. That is to say, Hypotheses 2–4 have been demonstrated.

Third, generation has a moderating effect on the relationship between workplace envy and regulatory focus. Specifically, “generation” has a positive moderating effect on the relationship between benign envy and promotion focus; therefore, the younger the employee’s generation, the more pronounced the effect of benign envy on promotion focus. On the other hand, generation has a positive moderating effect on the relationship between malicious envy and prevention regulatory focus; therefore, the older the employee’s generation, the more pronounced the effect of malicious envy on prevention regulatory focus. That is to say, all the relevant content of Hypothesis 5 can be demonstrated.

## 5. Discussion

### 5.1. Theoretical Contributions

First, workplace envy, as an emotional response frequently exhibited by employees during workplace comparisons, differentially influences knowledge-hiding behavior, depending on whether it is benign or malicious. This study pioneers a dual-path model that separately scrutinizes the intrinsic mechanisms through which these two distinct dimensions of envy affect knowledge-hiding behavior. Such a bifurcated examination is notably absent in prior literature. For instance, Peng et al. [11] probed the nexus between malicious envy and knowledge hiding but overlooked the potential interplay between benign envy and knowledge hiding. Yang and Tang [64], although they amalgamated both envy types into a unified model to assess individual behaviors, failed to delve into any one relationship with the granularity necessary for comprehensive understanding. Addressing this lacuna, our research bifurcates envy into two dimensions and delves deeply into their respective impacts on knowledge-hiding behavior. We aspire to uncover the underpinnings that bridge envy with knowledge hiding. Through our nuanced exploration of the differential influences of benign and malicious envy on knowledge hiding, we aim to enrich and extend the scholarly discourse on workplace envy.

Second, anchored in the foundations of social comparison and regulatory focus theories, this research delves into the intricate mechanisms through which employees’ workplace envy impacts knowledge-hiding behavior. Specifically, it verifies the mediating roles of both promotion and prevention regulatory focuses in the relationship between workplace envy and knowledge-hiding behavior. While Andiappan and Dufour [45] found a congruence between malicious envy and prevention regulatory focus and benign envy with promotion regulatory focus, our study embarks on an extended journey. We further decipher the knowledge-hiding behaviors triggered by these regulatory focuses under the aforementioned premise. Our findings reveal that workplace envy among employees does not inherently precipitate knowledge-hiding behaviors. Rather, distinct envy emotions catalyze different regulatory focuses, which in turn can either amplify or mitigate knowledge-hiding behaviors. This nuanced understanding transcends the traditionally linear perspective on the influence of workplace envy on knowledge hiding. Consequently, our research substantially augments the theoretical landscape related to knowledge-hiding behavior.

Third, building upon the framework of generation cohort theory, this study delves into generational distinctions in the way workplace envy influences knowledge-hiding behavior, aiming to unravel how employees from varied generational cohorts respond differently in analogous situations. In alignment with Su et al.’s [29] categorization approach for dissecting employee values, our research adopts 1992 as a demarcation point to differentiate between the “new generation” and the non-new generation, a method that resonates with contemporary Chinese generational research. From our bifurcated sample, it became evident that younger employee cohorts exhibited a more pronounced influence of benign envy on promotion regulatory focus. Conversely, older cohorts displayed a heightened effect of malicious envy on prevention regulatory focus. This exploration into generational nuances in relation to workplace envy’s influence on knowledge-hiding behavior significantly enriches the understanding of how generational cohort attributes intersect with employee work behavior.

### 5.2. Practical Significance

The research in this paper is of great relevance and includes three main aspects. First, knowledge hiding might offer fleeting advantages to individual employees, but its repercussions stifle the collective growth and innovative spirit of teams and organizations. It is incumbent upon organizations to not only promote but also incentivize knowledge sharing. By reinforcing organizational learning and robust knowledge management systems, an atmosphere conducive to open exchange can be nurtured. Even though knowledge sharing might seem above and beyond an employee’s designated role, its centrality in driving knowledge creation warrants the provision of apt rewards. Such tangible acknowledgments can serve as a potent deterrent against the inclination to hoard knowledge.

Second, envy in the workplace, be it benign or malicious, wields influence over employees’ knowledge-sharing tendencies. Recognizing this, it is crucial for organizational leaders to discern between these two facets of envy. Addressing them requires a dual strategy. Firstly, there is a need to channel malicious envy into its benign counterpart, mitigating its adverse impacts on work dynamics. Simultaneously, fostering an environment that supports and nurtures the aspirations of those with benign envy becomes imperative. Such an atmosphere not only satisfies their growth aspirations but also propels them to excel in their roles.

Third, diverse generational perspectives influence workplace behaviors, particularly around envy and knowledge sharing. With older cohorts or non-new-generation employees exhibiting tendencies to safeguard knowledge to uphold their stature, organizations face a unique challenge. Navigating this intricacy means that organizational strategies should prioritize these employees, cultivating an environment that encourages and rewards open knowledge dissemination.

### 5.3. Research Limitations

There are some limitations in the research process of this paper.

First, while the survey primarily relied on cross-sectional data, we collected data in two separate phases. However, these collection points were closely spaced. The variables, notably workplace envy and knowledge hiding, are sensitive in nature and may be susceptible to biases, particularly if participants’ emotions influenced their responses. Recognizing these potential limitations, future research could adopt a longitudinal approach, perhaps utilizing a diary study method, or incorporate additional experimental designs for more robust hypothesis testing, aiming to capture data that more accurately reflects reality.

Second, most of the study’s participants were from South China, although the research hypotheses were extensively tested. To enhance the generalizability of the results, future research should consider diversifying the geographical distribution of the sample.

Third, in this study, generational cohorts were categorized as “new generation” and “non-new generation” based on the division using the year 1992 as the demarcation point. It is noteworthy that existing literature on generational studies offers a variety of methods for dividing generational populations. In forthcoming research, alternative divisions could be considered, such as distinguishing cohorts into three or more generations or employing different temporal markers to enhance the depth and scope of relevant investigations.

## Figures and Tables

**Table 1:** Basic statistics.

Variables	Classification	Proportion	Variables	Classification	Proportion
Gender	Male	44.03%	Years of working	Less than 3 years	17.91%
	Female	55.97%		4 to 10 years	35.32%
Generation	New generation	46.52%		More than 10 years	46.77%
	Non-new generation	53.48%	Education level	Master’s degree and above	20.65%
Job class	Manager	39.30%		Bachelor degree	63.93%
	General employees	60.70%		Associate degree and below	15.42%

**Table 2:** Discriminant validity test (N = 402).

Model	χ^2^/df	RMSMA	SRMR	CFI	NFI	TLI	IFI
Five-factor model (BE;ME;PO;PE;NH)	2.233	0.055	0.053	0.926	0.874	0.921	0.926
Four-factor model (BE + ME;PO;PE;NH)	3.186	0.074	0.078	0.869	0.820	0.860	0.869
Four-factor model (BE;ME;PO + PE;NH)	3.436	0.078	0.073	0.854	0.806	0.844	0.854
Three-factor model (BE + ME;PO + PE;NH)	4.377	0.092	0.093	0.796	0.752	0.784	0.797
One-factor model (BE + ME + PO + PF + NH)	9.100	0.142	0.145	0.509	0.482	0.483	0.511

BE, benign envy; ME, malicious envy; PO, promotion regulatory focus; PE, prevention regulatory focus; and NH, knowledge hiding.

**Table 3:** AVE and CR values for each variable (N = 402).

Variable	AVE	CR
Benign envy	0.741	0.934
Malicious envy	0.582	0.874
Promotion regulatory focus	0.548	0.915
Prevention regulatory focus	0.502	0.900
Knowledge hiding	0.677	0.962

**Table 4:** Correlation matrix (N = 402).

Variables	1	2	3	4	5	6	7	8	9	10
1. Gender	1									
2. Job class	−0.004	1								
3. Years of working	−0.026	−0.018	1							
4. Education level	0.015	−0.023	−0.171 ***	1						
5. Generation	−0.013	−0.026	−0.665 ***	−0.048	1					
6. Benign envy	−0.044	−0.069	−0.05	−0.032	0.046	1				
7. Malicious envy	0.028	−0.002	0.153 **	−0.008	−0.164 ***	−0.514 ***	1			
8. Promotion regulatory focus	−0.068	0.042	−0.088	−0.025	0.148 **	0.596 ***	−0.587 ***	1		
9. Prevention regulatory focus	−0.003	0.003	0.066	0.03	−0.042	−0.600 ***	0.519 ***	−0.537 ***	1	
10. Knowledge hiding	0.043	−0.002	0.169 ***	0.092	−0.189 ***	−0.371 ***	0.358 ***	−0.375 ***	0.403 ***	1
Mean	1.560	1.393	2.289	1.948	0.535	2.503	3.566	2.540	3.685	3.118
S.D	0.497	0.489	0.752	0.599	0.499	1.007	0.994	0.891	0.819	1.108

** *p* < 0.01; *** *p* < 0.001.

**Table 5:** Main effect and mediating effects model test one (N = 402).

Variables	Knowledge Hiding	Promotion Regulatory Focus	Knowledge Hiding	Knowledge Hiding
	Model 1	Model 2	Model 3	Model 4
Gender	0.061	−0.085	0.044	0.042
Job class	−0.042	0.165 *	0.038	−0.005
Years of working	0.226 ***	−0.08	0.209 ***	0.208 ***
Education level	0.181 *	−0.023	0.183 *	0.175 *
Benign envy	−0.359 ***	0.596 ***		−0.227 ***
Promotion regulatory focus			−0.358 ***	−0.222 ***
$R^{2}$	0.173	0.367	0.172	0.204
Adjusted $R^{2}$	0.163	0.359	0.161	0.191
F	16.571 ***	45.886 ***	16.409 ***	16.902 ***

* *p* < 0.05; *** *p* < 0.001.

**Table 6:** Main effect and mediating effects model test two (N = 402).

Variables	Knowledge Hiding	Prevention Regulatory Focus	Knowledge Hiding	Knowledge Hiding
	Model 5	Model 6	Model 7	Model 8
Gender	0.072	−0.038	0.095	0.083
Job class	0.009	0.009	0.006	0.006
Years of working	0.184 **	−0.012	0.217 ***	0.187 **
Education level	0.196 *	0.054	0.179 *	0.180 *
Malicious envy	0.337 ***	0.521 ***		0.183 ***
Prevention regulatory focus			0.389 ***	0.296 ***
$R^{2}$	0.156	0.271	0.196	0.220
Adjusted $R^{2}$	0.146	0.262	0.186	0.208
F	14.680 ***	29.426 ***	19.324 ***	18.577 ***

* *p* < 0.05; ** *p* < 0.01; *** *p* < 0.001.

**Table 7:** Bootstrap test results of the mediation effect (N = 402).

Path	Benign Envy → Promotion Regulatory Focus → Knowledge Hiding	Malicious Envy → Prevention Regulatory Focus → Knowledge Hiding
Effect	−0.132	0.154
Boot SE	0.038	0.033
Boot LLCI	−0.213	0.095
Boot ULCI	−0.063	0.225

**Table 8:** The effects of benign envy on promotion regulatory focus between generation cohorts.

Variable	Promotion Regulatory Focus			
	Non-New Generation (N1 = 187)		New Generation (N2 = 215)	
	Model 9	Model 10	Model 11	Model 12
Gender	−0.158	−0.094	−0.108	−0.059
Job class	0.022	0.152	0.154	0.165
Years of working	−0.183	−0.06	0.103	0.115
Education level	−0.102	−0.079	0.059	0.136
Benign envy		0.483 ***		0.666 ***
$R^{2}$	0.016	0.228	0.012	0.487
$∆R^{2}$	0.016	0.206	0.012	0.474
F	0.734	10.670 ***	0.620	39.613 ***
Correlation coefficient between benign envy and promotion regulatory focus		0.477 ***		0.662 ***
Z-test	2.75 **			

** *p* < 0.01; *** *p* < 0.001.

**Table 9:** The effects of malicious envy on prevention regulatory focus between generation cohorts.

Variable	Prevention Regulatory Focus			
	Non-New Generation (N1 = 187)		New Generation (N2 = 215)	
	Model 13	Model 14	Model 15	Model 16
Gender	−0.062	−0.005	0.053	−0.026
Job class	0.09	0.060	−0.072	−0.043
Years of working	0.413 *	0.161	−0.004	−0.003
Education level	0.145	0.08	0.013	0.052
Malicious envy		0.661 ***		0.432 ***
$R^{2}$	0.037	0.415	0.002	0.193
$∆R^{2}$	0.037	0.378	0.002	0.191
F	1.734	25.673 ***	0.093	9.976 ***
Correlation coefficient between malicious envy and prevention regulatory focus		0.675 ***		0.430 ***
Z-test	3.57 ***			

* *p* < 0.05; *** *p* < 0.001.

## Data Availability

Not applicable.

## Appendix A

**Table A1:** Questionnaire.

**Envy** [60]
1. When I envy a colleague’s success, I think about how I can achieve the same success in the future
2. I want my better colleagues to lose their edge
3. If I find that my colleagues are better than me, I will try to make myself better
4. Envy of my colleagues’ performance or achievements motivates me to work hard to achieve my own goals
5. If a colleague has something I want to fight for, I want it from them
6. I feel uncomfortable with my jealous coworker
7. I strive to achieve better results like my colleagues
8. The feeling of envy makes me dislike other colleagues
9. If a colleague has outstanding abilities, outstanding achievements or great assets, I will strive to go after them
10. Seeing other colleagues make outstanding achievements makes me feel sick
**Regulatory focus** [61]
1. I focus on completing work tasks accurately to improve my job security
2. At work, I focus on completing the tasks assigned to me by my superiors
3. Completing work tasks is very important to me
4. At work, I try to fulfill the tasks assigned to me
5. At work, I focus on completing tasks that improve my job security
6. I will do my best to avoid mistakes in my work
7. Job security is an important factor for me when working online
8. I take care to avoid failure in my work
9. I am careful at work to avoid potential failure
10. I will try to seize opportunities at work to maximize my chances of promotion
I1 I tend to take risks at work to get ahead
12. If I have an opportunity to participate in a high-risk, high-reward project, I will take it
13. If there is no room for improvement in my job, I may look for a new one
14. The opportunity for growth is an important factor for me when looking for a job
15. I focus on completing tasks that will help me get promoted
16. I spend a lot of time thinking about how to achieve my ambitions
17. What I personally hope to achieve will affect my priorities
18. My ambition and pursuit motivate me to work
**Knowledge hiding** [14] (Suppose one of your colleagues asks you for help with something you know (something you know for sure)
1. I might act like I don’t know what he’s talking about
2. I may pretend I don’t know the information
3. I’ll probably say I don’t know. Though I know
4. I might say that I don’t know much about this topic
5. I may agree to help him. But I don’t really intend to do that
6. I may agree to help him, but I will give him different knowledge than he needs
7. I might tell him something else instead of what he really wants to know
8. I may tell him that I will help him later. But he’ll drag it out as long as he can
9 I may announce to him that my superiors do not allow me to share this knowledge with anyone
10. I’ll probably just tell him I won’t answer his question
11. I may explain that I am willing to chapter inform him. But someone doesn’t want me to
12. I may explain that this information is confidential and only accessible to those involved in a particular project
